# Scalp acupuncture for children with cerebral palsy: A protocol for a systematic review

**DOI:** 10.1097/MD.0000000000018062

**Published:** 2019-11-27

**Authors:** Go-Eun Lee, Pei-Ting Lee, Ni Ran, Jianwei Zhou

**Affiliations:** a School of Acupuncture – Tuina and Rehabilitation, Chengdu University of Traditional Chinese Medicine, Chengdu; b Southwest Medical University, Luzhou; c Sichuan Academy of Chinese Medicine Sciences, No. 51, Fourth Section of Ren-min South Road, Chengdu 610075, Sichuan Province, PR China.

**Keywords:** cerebral palsy, children, protocol, scalp acupuncture, systematic review

## Abstract

**Background:**

Cerebral palsy (CP) describes a group of permanent disorders of movement and posture causing activity limitations, leading the most common movement disorder to children. Scalp acupuncture (SA) is one of several specialized acupuncture techniques, and it has been used widely in China to alleviate several CP symptoms, despite the deficiency of high-quality evidence related to this practice. Therefore, we plan to conduct a protocol of systematic review aimed at systematically reviewing all the clinical evidence on the effectiveness of scalp acupuncture for treating CP in children.

**Methods:**

The following electronic databases will be searched from inception to May 1, 2019 MEDLINE, PubMed, Web of Science, EMBASE, the Cochrane Central Register of Controlled Trials (Cochrane Library), Chinese National Knowledge Infrastructure (CNKI), Chinese Scientific Journals Database (VIP), Wan-fang Database, and Chinese Biomedical Literature Database (CBM). All published English and Chinese articles randomized controlled trials (RTCs) will be included. All types of CP of children in the trials will be included in this study and these individuals will be involved as core searchers to evaluate the efficacy of scalp acupuncture. Rev Man V.5.3 software will be implemented for the assessment of bias risk, data synthesis, subgroup analysis, and meta-analyses if inclusion conditions are met. Continuous outcomes will be presented as mean difference (MD) or standard mean difference (SMD), while dichotomous data will be expressed as a relative risk.

**Results:**

The systematic review will synthesize the available knowledge surrounding scalp acupuncture for children with CP. The findings will be synthesized to determine the efficacy and safety of scalp acupuncture for children with CP.

**Conclusion:**

This protocol will present the evidence of whether scalp acupuncture is an effective intervention for children with CP.

## 1 Introduction

Cerebral palsy describes a heterogeneous group of permanent disorders of movement and posture which are attributed to non-progressive disturbances in the developing fetal or infant brain and cause limitations inactivity.^[1]^ The motor disorders of cerebral palsy are often accompanied by disturbances of sensation, perception, cognition, communication, and behavior, epilepsy, and secondary musculoskeletal problems.^[2]^ In the second half of the past century, most reviews concluded that the prevalence of cerebral palsy (CP) in industrialized nations was fairly stable at 1.5–2.5 cases per 1000 live births, but with a modest increase in the last two decades of the 20th century owing largely to the greatly increased survival of very premature infants as a result of the success of the new technology.^[3,4]^

CP is a heterogeneous and poorly understood disorder with no cure. Medical costs for individuals with CP are estimated nearly $1.2 million. Over 10 years, total Cerebral Palsy Alliance Research Foundation philanthropic funding was $21.9 million, including people, infrastructure, strategic and project support.^[5–7]^

The cause and mechanism of cerebral palsy has been studied and debated for more than 100 years, but exact cause is still unclear. Several risk factors are involved in the development of cerebral palsy, which can occur during pregnancy, at birth, or after birth. These include maternal infections, premature birth, low birth weight, disruption of oxygen or blood supply to the brain, and infections or injury in the neonatal period and in early childhood,^[8]^ resulting in abnormalities in brain development that lead to cerebral palsy, although risk factors have been identified, but they overlap and interact with each other in ways that are not easy to dissect.^[9]^ There are several different types of cerebral palsy, including spastic, dyskinetic, ataxic, hypotonic, and mixed. Most (77.4%) of the children identified with CP had spastic CP.^[10]^ Cerebral palsy is a chronic condition with no effective cure and as such the overall goal of treatment is to improve quality of life and participation in life situations. The management involves neurological rehabilitation and management of co-morbidities.^[11,12]^ Medical management involves care from primary-care physicians with input from specialists in neurology, orthopaedics, and rehabilitation medicine. Physicians should also work in conjunction with rehabilitation therapists, educators, nurses, social care providers, and school teachers.^[13]^

Scalp acupuncture is an important means of rehabilitation of cerebral palsy in traditional Chinese medicine. It is a contemporary acupuncture technique integrating traditional Chinese needling methods with Western medical knowledge of representative areas of the cerebral cortex.^[14,15]^ Modern medical research suggests that scalp acupuncture can dilate cerebral cortical blood vessels, improve the blood supply of diseased cortex, promote the metabolism of brain cells and the expression of neurotrophic factors, and specifically activate specific motor function areas of cerebral cortex.^[16,17]^ Several studies have evaluated scalp acupuncture as a treatment for CP,^[18–20]^ but the lack of evidence makes alternative medicine difficult to implement in practice. Thus, the goal of this systematic review is to estimate the effectiveness and safety of acupuncture on CP.

## 2 Methods

### 2.1 Inclusion creteria for study selection

#### 2.1.1 Types of studies

All available randomized controlled trials (RCTs) and quasi-randomized controlled clinical trials (quasi-RCTs) on scalp acupuncture for children with CP will be included. Others such as retrospective study, case report, and studies which use inappropriate random sequence generation methods will be excluded. Language will be restricted to Chinese and English.

#### 2.1.2 Types of participants

Studies involve participants with a diagnosis of CP. There were no limitations of age, sex, or race. We excluded trials that included participants suffering from other serious illness such as cancer, liver disease, or kidney disease.

#### 2.1.3 Types of interventions and types of comparisons

We will include studies using several types of scalp acupuncture (Standard Nomenclature SA, Jin Three-Needle Therapy, Zhu shi, Jiao shi, Fang shi, and Tang shi SA) or scalp acupuncture combined with rehabilitation as experimental interventions. The intervention of controlled group can be conventional therapy (CT) included drugs, and physical, exercise, occupational, language therapy interventions.

#### 2.1.4 Types of outcome measures

Primary outcomes will include 88 items of gross motor function scale (GMFM-88), the Modified Ashworth Scale and the Activity of Daily Living Scales (ADL).

Secondary outcomes will include total effective rate, motor function improvement and adverse events caused by scalp acupuncture (SA), such as dizziness, nausea, vomiting.

### 2.2 Search methods for identification of studies

#### 2.2.1 Electronic searches

Relevant databases include: MEDLINE, PubMed, Web of Science, EMBASE, the Cochrane Central Register of Controlled Trials (Cochrane Library), Chinese National Knowledge Infrastructure (CNKI), Chinese Scientific Journals Database, Wan fang Database, and Chinese Biomedical Literature Database (CBM). All English and Chinese RCTs published in electronic databases from inception to May 2019 will be included in this review. The search strategy will be formulated in accordance with the guidance provided by the Cochrane Handbook. The Medline search strategy is listed in Table 1, which includes all search terms, and other searches will be conducted based on these results.

**Table 1:**
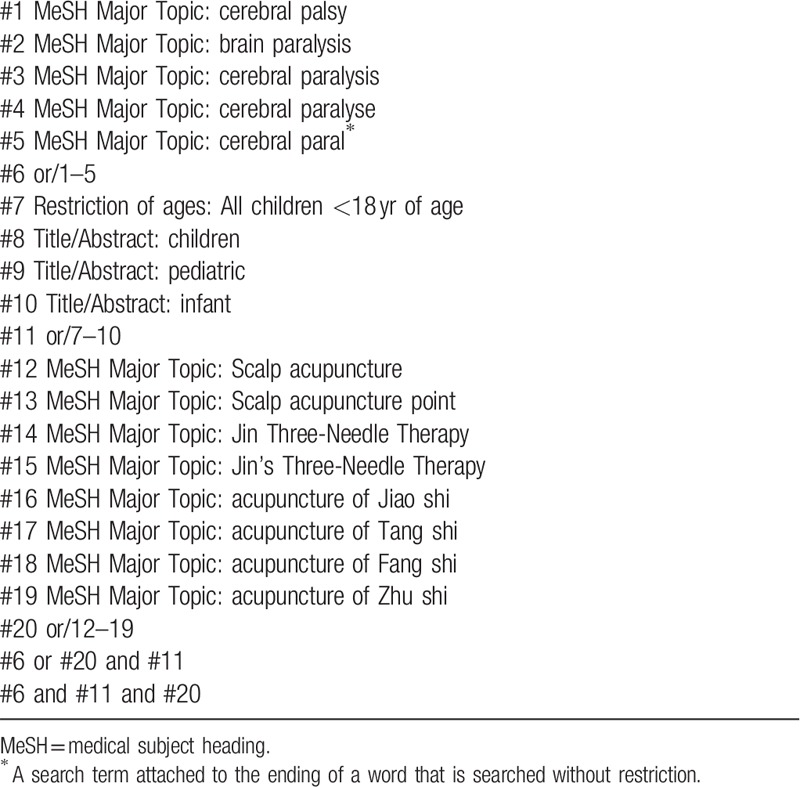
Medline search strategy.

#### 2.2.2 Searching other resource

Relevant systematic review or meta-analysis of RCTs will be electronically searched. Moreover, we will filter relevant medical journals and magazines to identify literature which is not included in the electronic databases.

### 2.3 Data collection and analysis

#### 2.3.1 Selection of studies

Depending on the type of literature research, intervention measures, and object of study, 2 authors GEL and PTL will rule out certainly not related literatures, respectively, by reading the title and abstract in early screening and collect all possible relevant and certain related researches. We will contact corresponding author if the information of article is incompletion. Having disagreements, we plan to consult experts.

#### 2.3.2 Assessment and quality of included studies

Authors GEL, NR, and PTL will evaluate quality of articles and assess the risk of bias based on the domains and criteria of the Cochrane Collaboration's tool.^[21]^ Controversial problems of article will consult experts to solve. Assessment and quality of included studies include that what randomized method has been used, whether the results of the data are complete and whether grouping scheme is hidden or not.

#### 2.3.3 Data extraction

Authors (GEL and NR) plan to extract data of articles and to solve different ideas by discussing with experts. The datum of each selected trial will be extracted and an electronic form will be recorded that includes that basic information of studies (numbering, which combines the first author's last name with year published, the contact information of corresponding author), sample size and grouping method, object of study characteristics, which will express as mean addition and subtraction standard deviation and percentage, and details of intervention methods, including treatment time, selection of acupoints, data type of treatment efficacy, treatment cycles, side effects, and follow-up.

#### 2.3.4 Measures of treatment effect

A risk ratio (RR) with 95% confidence intervals (95% CIs) will be presented for dichotomous variables. Mean difference (MD) or standard mean difference (SMD) with 95% CI will be presented for continuous outcomes. Other binary data will be changed into the RR form.

#### 2.3.5 Dealing with missing data

In view of the lack of data that needs to be extracted in the included document, if there are a statistical missing data, we will attempt to contact the authors by phone or email. If the missing data are not obtained, the available data will be analyzed with the assumption that it is missing at random. If necessary, we will impute missing data using replacement values.

#### 2.3.6 Assessment of heterogeneity

The heterogeneity of each study will be analyzed using *χ*^2^ test and *P* values, and the heterogeneity will be evaluated by I2 statistic. An interpretation of I2 is as follows: I2 ≥ 50% will be considered as representing substantial heterogeneity, while I2 < 50% will be taken as evidence of no heterogeneity. If the clinical and methodological heterogeneity are not found, the stochastic effect model will be assessed by merger analysis. Only descriptive analysis will be performed when the heterogeneity is oversize.

#### 2.3.7 Assessment of reporting bias

We will draw support from funnel plot and statistical test to determine reporting bias.

#### 2.3.8 Data synthesis

From the aspect of clinical research, we will consider whether the meta-analysis is carried out. The clinical research includes the research designing of measurement methods, intervention methods, the length of treatment, and whether the choice of the control group is same to determine. When a couple of good multiple homogeneity studies are included, we will perform meta-analyses with Review Manager 5.3 When I2 < 50%, the fixed effect model will be selected and the random-effect model will be selected I2 > 50%. If not, we will fail to implement meta-analysis.

#### 2.3.9 Other analysis

If the heterogeneity is caused by clinical trials mentioned above, subgroup analysis will be conducted, and according to the outcomes of data synthesis, detailed subgroup will be classified. If we identify substantial heterogeneity, the following subgroup analyses plan to carry out.

(1) Different types of SA therapies
(2) Types of CP (disorder of motor function, disorder of intellectual, etc)

Sensitivity analysis: By changing the genre of research (incorporating or excluding a particular study) and reanalysis of simulated missing data, we will observe fluctuation of termination.

#### 2.3.10 Assessment of publication bias

We will draw support from funnel plots when studies are more than 10 trials. If the points of the funnel plot are dispersed and asymmetrical, we will consider that the reporting bias is existing and the reliability is low. In the opposite condition the reporting bias will be considered as non-existent and the result is reliable.

## 3 Discussion

Cerebral palsy is a permanent non-progressive cerebral lesion, which is one of the most severe brain diseases that a child can have,^[22]^ and has a high disability rate, which can cause physical and emotional burden and a possible decrease in the quality of life. The goal of cerebral palsy treatment is to help children work on their own, improving their quality of life. Also, treatment measures should be determined based on scientific evidence according to the growth cycle of differences in age of persons. NOVAK studies about best-available interventional evidence for children suffering from cerebral palsy.^[23]^ Green interventions included botulinum toxin, anticonvulsants, bimanual training, casting, home programmes, bisphosphonates, constraint-induced movement therapy, context-focused therapy, diazepam, fitness training, hip surveillance and so on but scalp acupuncture was not included among the 16 “do it” treatments. Scalp acupuncture is one of several specialized acupuncture techniques. According to the current evidence, SA therapy for ischemic and hemorrhagic stroke has been empirically established and is used worldwide.^[24,25]^ SA as a monotherapy or as adjunctive therapy to rehabilitation treatment might have benefits for treating CP.

However, due to the small number of included studies, the lack of appropriate sample size, poor methodological qualities, and low quality of evidence for main findings, the findings of this review should be interpreted with great caution. Larger and more rigorous high-quality RCTs should be performed in this area.

## Author contributions

**Conceptualization:** Jianwei Zhou, Goeun Lee.

**Data curation:** Ni Ran.

**Methodology:** Peiting Lee.

**Writing – original draft:** Goeun Lee, Peiting Lee.

**Writing – review & editing:** Goeun Lee, Jianwei Zhou.
